# Complications of Cochleostomy Versus Round Window Surgical Approaches: A Systematic Review and Meta-Analysis

**DOI:** 10.7759/cureus.25451

**Published:** 2022-05-29

**Authors:** Vardhan S Avasarala, Sanjay K Jinka, Anita Jeyakumar

**Affiliations:** 1 Otolaryngology - Head and Neck Surgery, Northeast Ohio Medical University, Rootstown, USA; 2 Otolaryngology - Head and Neck Surgery, Mercy Health, Youngstown, USA

**Keywords:** scala tympani insertion, speech perception, vestibular dysfunction, electrical impedance, electrode-to-modiolus distance, scalar shift, hearing preservation, cochlear implant, round window, cochleostomy

## Abstract

We conducted a systematic review and meta-analysis to compare round window (RW) and cochleostomy (C) surgical approaches for the placement of cochlear implants (CIs). After obtaining the Institutional Review Board (IRB) approval, 213 peer-reviewed articles published between January 1, 2000, and August 1, 2021, comparing RW and C approaches were identified via a search on Google Scholar, Cochrane, and PubMed. The inclusion criteria were articles having an English version and involving only human subjects (cadaveric or alive). Statistical analysis of compiled electrode-to-modiolus distances was performed with two-sample independent t-tests. Live patients were categorized as having complete hearing preservation (<10 dB threshold shift), partial hearing preservation (10-20 dB shift), or minimal hearing preservation (>20 dB shift). Chi-squared testing was used to compare the distribution of hearing preservation categories between surgical approaches. Due to the heterogeneous nature of the data, only summative information was provided on the effects of approaches on trauma, electrical impedance, speech perception, vestibular dysfunction, ease of scala tympani insertion, and scalar shift. A total of 3,797 CI patients were evaluated. The RW approach resulted in a smaller (0.15 mm smaller on average, p<0.05) electrode-to-modiolus distance when compared to the C approach. The RW approach (93.0%) led to statistically better hearing preservation than the C approach (84.3%) (p<0.05). The RW approach was also associated with better outcomes in terms of speech perception, ease of scala tympani insertion, and reduced scalar shift. No difference between approaches was found with regard to trauma, electrical impedance, and vestibular dysfunction. Based on our findings, the RW approach appears to have several benefits compared to the C approach.

## Introduction and background

A cochlear implant (CI) is a device used to restore hearing in patients who cannot be helped with a hearing aid [1]. CIs have two components: an internal and an external portion. Unlike hearing aids that amplify sound, a CI transmits sound by directly stimulating the auditory nerve via the cochlear modiolus [2]. Two main surgical approaches are used to insert CI electrodes within the cochlea: cochleostomy (C) and round window (RW) [3]. The C approach involves placing the electrode of the CI into the scala tympani by creating an opening anterior and inferior to the RW [4,5]. On the other hand, the RW approach involves inserting this electrode via the RW itself, with or without drilling the edges of the window [6]. Some authors term drilling edges of the RW as an “extended round window”; however, in the present study, all RW insertions, irrespective of window enlargement/removal of annular bone, are categorized as RW approaches.

Surgeons continue to debate as to which technique is superior. Even though some systematic reviews/meta-analyses on the topic are available in the literature, there are many outcomes to consider when comparing the two surgical approaches, including trauma, hearing preservation, and speech perception.

We conducted a systematic review to compare surgical approaches in the context of trauma, electrical impedance, speech perception, vestibular dysfunction, ease of scala tympani insertion, and scalar shift. In the context of this review, we carried out statistical testing of homogenized data to identify which surgical approach yields better hearing preservation and electrode-to-modiolus distance.

## Review

Methods

Ethical Approval

Institutional Review Board (IRB)-exempt status was obtained from our institution to conduct this review.

Literature Search

**Figure 1:**
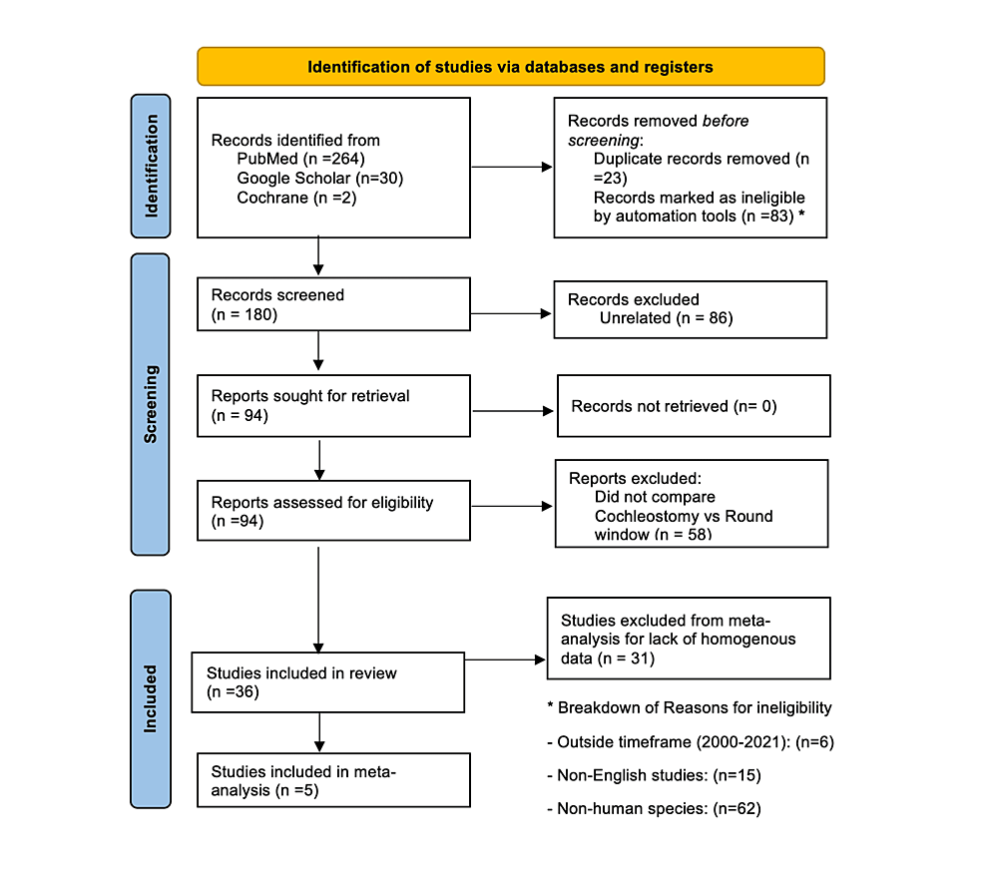
PRISMA flow diagram PRISMA: Preferred Reporting Items for Systematic Reviews and Meta-analyses

A systematic review of available literature on Cochrane, PubMed, and Google Scholar published between January 1, 2000, and August 1, 2021, was conducted. The methodology is summarized in Figure 1. A total of 213 peer-reviewed articles comparing RW and C approaches were identified. The inclusion criteria were as follows: articles having an English version and involving only human subjects. Human subjects included both cadaveric and live subjects. Peer-reviewed articles were identified from PubMed, Google Scholar, and Cochrane by employing the following broad Medical Subject Headings (MeSH) search: ("round window") AND ("cochleostomy"). Quality and validity were determined as per the Preferred Reporting Items for Systematic Reviews and Meta-analyses (PRISMA) guidelines (Figure 1).

Electrode-to-Modiolus Distance Analysis

For the analysis of electrode-to-modiolus distance, the only literature available for review used the Med-EL standard 12-electrode arrays, and hence only those papers including data about Med-EL standard 12-electrode contact arrays were specifically included. Further, all included studies had to determine the electrode-to-modiolus distance in a consistent manner, specifically via combined flat-panel CT (FPCT) and curved multiplanar reconstruction (MPR). In total, this approach yielded two papers for analysis [4,7].

Extracted data were made comparable by analyzing RW and C approach electrode-to-modiolus distances for the 12-electrode positions common to all studies. Electrode distances were pooled and compared with two-sample independent t-testing.

Hearing Preservation Analysis

For the analysis of hearing preservation, only those papers that reported hearing preservation in decibels were included. Although 18 papers reviewed hearing preservation, not every paper reported preservation in decibels. In total, our inclusion criteria yielded three papers for analysis [8-10].

Extracted decibel readings were pooled and used to classify patients into the following categories: patients having complete hearing preservation (mean hearing threshold increasing ≤10 dB), those with partial preservation (increasing between 10 dB and 20 dB), and patients with minimal preservation to complete loss of residual hearing (increasing ≥20 dB). A chi-squared test was used to compare the distribution of hearing preservation categories between the two surgical approaches.

Other Analyses

Eight papers were analyzed for ease of scala tympani insertion [4,7,11-16]. Two papers were analyzed for scalar shift [17,18]. Three papers were analyzed for electrical impedance [19-21]. Seven papers were analyzed for intraoperative trauma [5,6,22-26]. Five papers were analyzed for speech perception [21,27-30]. Three papers were analyzed for vestibular dysfunction [31-33]. Due to the lack of homogeneity in the data, statistical analyses could not be carried out, and summative data was reported.

Results

Sample Size and Demographics

In total, 36 studies met our criteria for inclusion (Figure 1). The included papers evaluated outcomes and characteristics of 3,797 cadaveric and live human CIs. Twenty-nine and 7 papers were regarding live human and cadaveric subjects, respectively. Of the 29 studies on live humans, the average patient age was 38.4 years. The average patient age in the studies ranged from 2.4 to 75.5 years. A breakdown based on sex and race could not be provided as some papers did not include specific information about the sex or race of the patients.

Electrode-to-Modiolus Distance Analysis

Analyzing the two papers regarding electrode-to-modulus distance yielded 48 electrode arrays with a mean patient age of 26 years. Patient ages ranged from 0.8 to 64 years. Of note, 60% of the patients were male.

**Figure 2:**
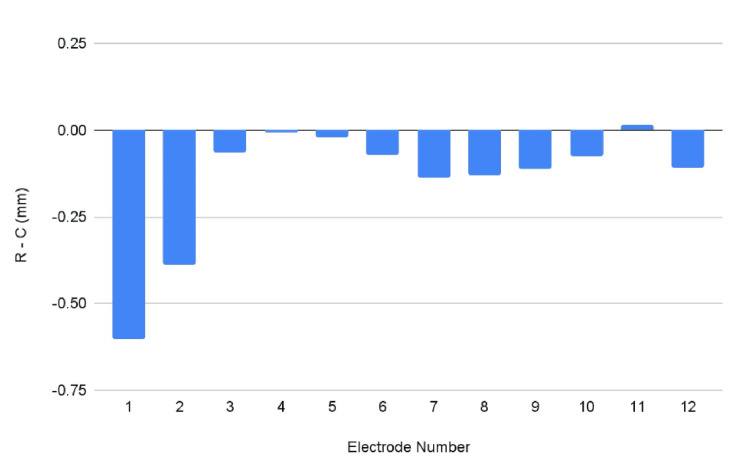
Average difference between round window and cochleostomy surgical approaches in terms of electrode-to-modiolus distance at electrodes 1-12

Two-sample independent t-testing revealed that the RW approach provided a significantly smaller electrode-to-modiolus distance when compared to the C approach. Specifically, the distance was 0.15 mm smaller on average with significant differences favoring the RW approach identified in electrodes 1-3, 5-10, and 12. No significant difference was found in electrodes 4 or 11 (Table 1, Figure 2).

**Table 1:** Average difference between round window and cochleostomy surgical approaches in terms of electrode-to-modiolus distance at electrodes 1-12 *Significant difference between surgical approaches, p<0.05

	Electrode #					
	1	2	3	4	5	6
R - C (mm)	-0.603*	-0.390*	-0.063*	-0.006	-0.020*	-0.072*
	Electrode #					
	7	8	9	10	11	12
R - C (mm)	-0.138*	-0.129*	-0.112*	-0.077*	0.015	-0.109*

Hearing Preservation Analysis

Each of the three studies evaluated for hearing preservation used low frequency-pure tone averages (PTA) to assess residual hearing loss. Two of the studies measured hearing preservation with PTA thresholds between 125 and 500 Hz. The third study measured hearing preservation with PTA thresholds at 250, 500, and 1000 Hz.

Of the patients analyzed for hearing preservation, 141 and 115 underwent the RW and C approaches, respectively. One of the studies included was a systematic review from 2013 and did not specify the average age of the participants. The remaining two studies included in the analysis were from 2014 and were not included in this systematic review. These latter studies had an average patient age of 42 years.

**Figure 3:**
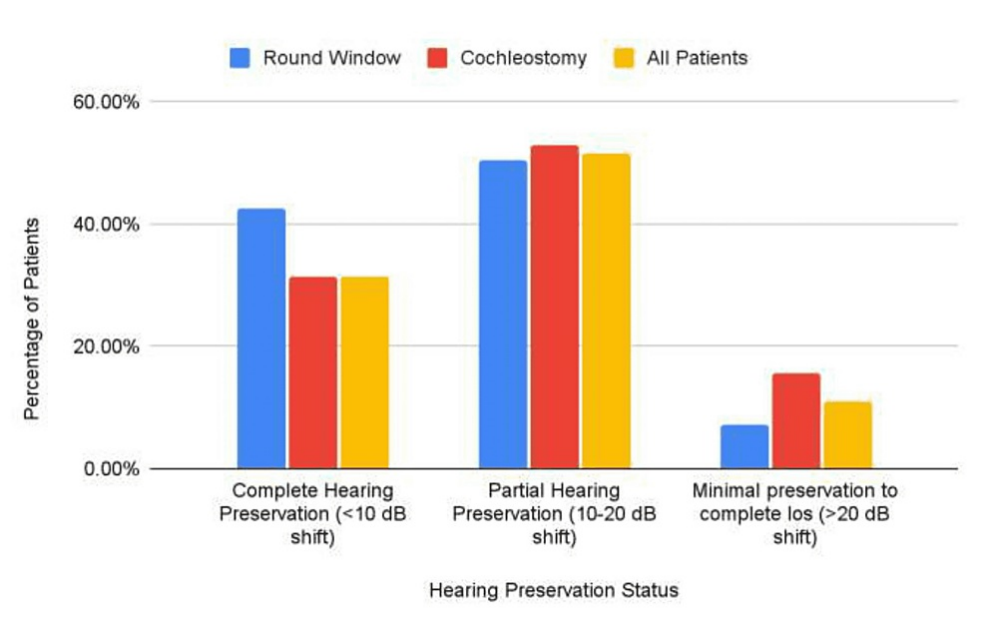
Comparison of hearing preservation between the round window and cochleostomy surgical approaches

Of the RW-approach patients, 93% (n=77) had less than a 20-dB shift in their hearing threshold with 7.1% (n=10) having greater than a 20-dB shift. Of the C-approach patients, 84.3% (n=36) had less than a 20-dB shift in their hearing threshold with 15.7% (n=18) having greater than a 20-dB shift (Table 2, Figure 3).

**Table 2:** Comparison of hearing preservation between the round window and cochleostomy surgical approaches

Hearing preservation	Surgical approach		
	Round Window	Cochleostomy	All patients
Complete hearing preservation (<10 dB shift)	42.6% (6)	31.3% (36)	31.3% (96)
Partial hearing preservation (10-20 dB shift)	50.4% (71)	53.0% (132)	51.6% (132)
Minimal preservation to complete loss (>20 dB shift)	7.1% (10)	15.7% (28)	10.9% (28)
Total	100% (141)	100% (115)	100% (256)

A chi-squared test revealed a significant (p<0.05) difference between these distributions, suggesting that the RW approach leads to significantly better outcomes with respect to hearing preservation.

Trauma

**Figure 4:**
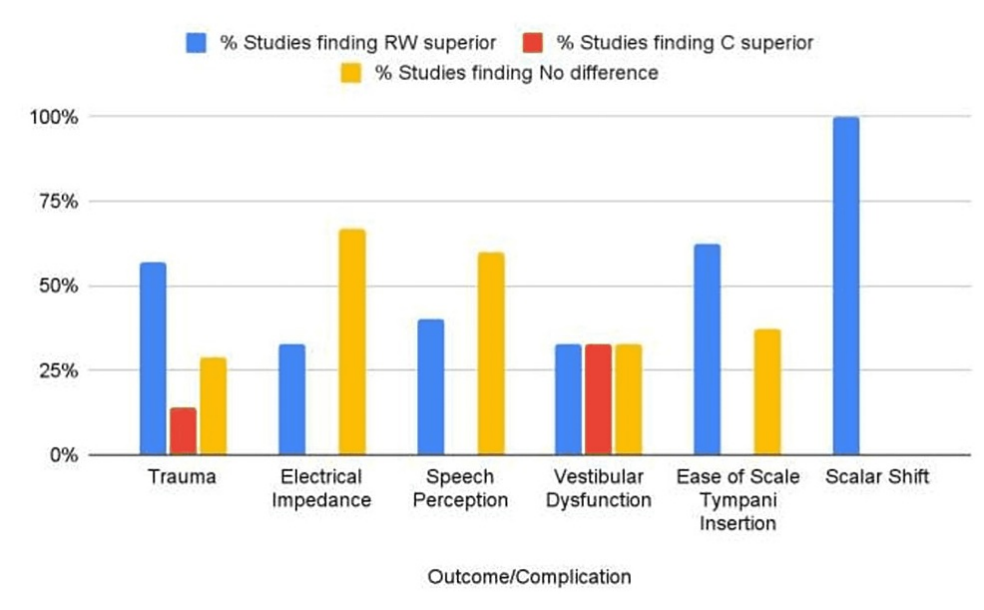
Percentage of studies that indicate the superiority of RW or C approaches regarding trauma, electrical impedance, speech perception, vestibular dysfunction, ease of scala tympani insertion, and scalar shift RW: round window; C: cochleostomy

Seven of the included studies evaluated intraoperative trauma [5,6,22-26]. Four studies showed that the RW approach yielded less intracochlear trauma when compared to the C approach [5,6,24,26]. Two studies [22,25] showed no difference, and one study [23] showed that the C approach yielded less intracochlear trauma when compared to the RW approach (Table 3, Figure 4).

**Table 3:** Percentage of studies that indicate the superiority of RW or C approaches regarding trauma, electrical impedance, speech perception, vestibular dysfunction, ease of scala tympani insertion, and scalar shift RW: round window; C: cochleostomy

Outcome/complication	Number of studies	% of studies finding RW superior	% of studies finding C superior	% of studies finding no difference
Trauma	7	57% (4)	14% (1)	29% (2)
Electrical impedance	3	33% (1)	0% (0)	67% (2)
Speech perception	5	40% (2)	0% (0)	60% (3)
Vestibular dysfunction	3	33% (1)	33% (1)	33% (1)
Ease of scala tympani insertion	8	62.5% (5)	0% (0)	37.5% (3)
Scalar shift	2	100% (2)	0% (0)	0% (0)

Electrical Impedance

Three of the included studies evaluated electrical impedance [19-21]. One study [19] showed that the RW approach yielded lower electrical impedance when compared to the C approach while two studies [20,21] indicated that there was no difference (Table 3, Figure 4).

Speech Perception 

Five of the included studies evaluated speech perception [21,27-30]. Two studies [28,30] showed that the RW approach resulted in improved speech perception when compared to the C approach while three studies [21,27,29] indicated that there was no difference (Table 3, Figure 4).

Vestibular Dysfunction 

Three of the included studies evaluated vestibular dysfunction [31-33]. One study showed that the RW approach resulted in less vestibular dysfunction when compared to the C approach [32]. One study [33] showed that the C approach led to less vestibular dysfunction when compared to the RW approach while the last study [31] indicated that there was no difference (Table 3, Figure 4).

Ease of Scala Tympani Insertion

Eight of the included studies evaluated the ease of insertion into the scala tympani [4,7,11-16]. Five studies indicated that the RW approach was better for insertion into the scala tympani when compared to the C approach [4,11,12,15,16]. The remaining three studies indicated that there was no difference (Table 3, Figure 4) [7,13,14].

Scalar Shift

Two of the included studies evaluated the presence of scalar shift [17,18]. Both studies indicated that the RW approach yielded less scalar shifting when compared to the C approach (Table 3, Figure 4).

Discussion

Hearing preservation is an integral outcome to consider when determining which surgical approach to use. Our data suggest that the RW approach leads to optimum hearing preservation in pediatric and adult populations. While this may lead surgeons to consider the RW over the C approach, it is also important to understand other differences in outcomes between these approaches.

Electrode-to-modiolus distance is a crucial consideration. The importance of electrode-to-modiolus distance remains controversial, but decreased distance appears to be associated with decreased current levels for suprathreshold simulation [34,35]. Although clinical implications of this finding remain unclear, one potential implication is slowed consumption of implant batteries. Our study shows that the RW approach leads to decreased electrode-to-modiolus distance when compared to the C approach. In patients where battery life is a serious consideration, this may be worth thinking about. However, the importance of this finding will most likely become clearer as further studies on the topic are completed.

A more clinically useful outcome that we evaluated was intraoperative trauma to the cochlea. Minimizing intracochlear trauma is pivotal for residual hearing preservation [36]. Our analysis suggests that the RW approach may lead to less trauma than the C approach (Table 3). This makes intuitive sense as the RW approach does not require the creation of a separate opening for the insertion of the CI. However, for surgeons who are more experienced in the C approach, or in cases of challenging anatomy, an RW enlargement may be necessary. This increases the risk for potential trauma, and all things considered, we cannot recommend one surgical approach over the other for minimizing trauma specifically.

Electrical impedance is a measure indicative of the integrity and function of electrodes. It reflects the status of the tissue-to-electrode interface [21]. Lower electrical impedance correlates with higher integrity of the implant. Our analysis did not reveal any superiority of either the RW or C approach in reducing electrical impedance with two-thirds of the studies we reviewed regarding electrical impedance revealing no difference between the approaches [20,21]. The remaining study found that the RW approach was preferable [19]. Despite this, we cannot recommend one surgical approach over the other for minimizing electrical impedance, but future meta-analysis/systematic review may shed more light on the potential benefits of the RW approach.

Speech perception is a measure of CI integrity. Speech perception is defined as the ability to identify and understand human speech and discriminate between monosyllabic words. Our analysis did not reveal the superiority of either the RW or C approach in maximizing speech perception with 60% of the studies we reviewed regarding speech perception revealing no difference between the approaches [21,27,29]. The remaining 40% of studies revealed that the RW approach was superior, suggesting that the RW approach may maximize speech perception when compared to the C approach [28,30]. Further systematic reviews/meta-analyses of this topic are necessary to better understand this relationship.

Vestibular dysfunction and dizziness are common side effects associated with CIs [31]. Our analysis did not exhibit the superiority of either the RW or C approach in minimizing vestibular dysfunction with one-third of the studies we reviewed finding the RW approach superior, one-third finding the C approach superior, and one-third finding neither approach superior [31-33]. These contradictory findings mean that we cannot recommend one surgical approach over the other for minimizing vestibular dysfunction specifically.

Ease of scala tympani insertion is critical as patients with electrodes in the scala tympani experience better overall hearing outcomes, less postoperative vertigo, and preservation of residual hearing when compared to those with electrodes in the scala vestibuli [37-42]. Our analysis revealed that 62.5% of studies indicated the superiority of the RW approach for ease of insertion [4,11,12,15,16]. The remaining 37.5% of studies indicated no difference between the RW and C approaches [7,13,14]. Ease of insertion is largely subjective and dependent on a host of factors, including patient anatomy and surgeon skill in addition to the surgical approach. However, the lack of studies supporting the C approach coupled with the fact that a majority of papers favor the RW approach leads us to recommend the RW approach to maximize the ease of insertion into the scala tympani.

The scalar shift is a strongly unwanted complication in CI surgery, which results in damage to the cochlear microstructure with loss of hair cells and spiral ganglion neurons [40,43]. Preventing this complication is of critical importance. Our analysis finds that all of the included studies evaluating scalar shift identify the RW approach as the preferred surgical approach for minimizing this complication [17,18]. For this reason, we strongly recommend the RW approach when striving to minimize scalar shift.

Bias

The results of this systematic review may be limited by the innate risk of selection bias, implementation bias, as well as attribution bias associated with systematic reviews.

Level of evidence

The studies included in the meta-analysis had a level of evidence of 2. 

Limitations

Our review is limited primarily by the lack of homogenous data and small sample size, meaning that we were able to perform statistical analysis on only two of the eight desired surgical outcomes/complications. Furthermore, there is no consensus as to which variables carry the most weight when determining which surgical approach is better, and hence we assigned equal weight to all variables.

## Conclusions

Our meta-analysis revealed that when compared to the traditional C approach, the RW approach for the placement of cochlear implants is associated with better hearing preservation and reduced electrode-to-modiolus distance. The clinical significance of the reduced electrode-to-modiolus distance is yet to be elucidated, but the reduced distance may correlate with longer-lasting batteries in these devices.

Our systematic review indicated that the RW approach can lead to better outcomes of speech perception, more ease of scala tympani insertion, and reduced scalar shift. Regarding trauma, electrical impedance, and vestibular dysfunction, other factors like surgeon experience and patient anatomy may be more predictive of success than the choice of surgical approach. Taken together, we recommend the RW approach over the C approach for new surgeons without a preferred operative approach seeking to optimize patient outcomes and ease of insertion. We believe further studies can reveal other differences in short- and long-term outcomes between these approaches.

## References

[1] Deep NL, Dowling EM, Jethanamest D, Carlson ML (2019). Cochlear implantation: an overview. J Neurol Surg B Skull Base.

[2] (2022). Cochlear implants. https://www.nidcd.nih.gov/health/cochlear-implants.

[3] Mangus B, Rivas A, Tsai BS, Haynes DS, Roland JT Jr (2012). Surgical techniques in cochlear implants. Otolaryngol Clin North Am.

[4] Jiam NT, Jiradejvong P, Pearl MS, Limb CJ (2016). The effect of round window vs cochleostomy surgical approaches on cochlear implant electrode position: a flat-panel computed tomography study. JAMA Otolaryngol Head Neck Surg.

[5] Richard C, Fayad JN, Doherty J, Linthicum FH Jr (2012). Round window versus cochleostomy technique in cochlear implantation: histologic findings. Otol Neurotol.

[6] Roland PS, Wright CG, Isaacson B (2007). Cochlear implant electrode insertion: the round window revisited. Laryngoscope.

[7] Fan X, Xia M, Wang Z (2018). Comparison of electrode position between round window and cochleostomy inserting approaches among young children: a cone-beam computed tomography study. Acta Otolaryngol.

[8] Havenith S, Lammers MJ, Tange RA, Trabalzini F, della Volpe A, van der Heijden GJ, Grolman W (2013). Hearing preservation surgery: cochleostomy or round window approach? A systematic review. Otol Neurotol.

[9] Nordfalk KF, Rasmussen K, Bunne M, Jablonski GE (2016). Deep round window insertion versus standard approach in cochlear implant surgery. Eur Arch Otorhinolaryngol.

[10] Sun CH, Hsu CJ, Chen PR, Wu HP (2015). Residual hearing preservation after cochlear implantation via round window or cochleostomy approach. Laryngoscope.

[11] O'Connell BP, Cakir A, Hunter JB (2016). Electrode location and angular insertion depth are predictors of audiologic outcomes in cochlear implantation. Otol Neurotol.

[12] Wanna GB, Noble JH, Carlson ML (2014). Impact of electrode design and surgical approach on scalar location and cochlear implant outcomes. Laryngoscope.

[13] Nguyen S, Cloutier F, Philippon D, Côté M, Bussières R, Backous DD (2016). Outcomes review of modern hearing preservation technique in cochlear implant. Auris Nasus Larynx.

[14] Hassepass F, Aschendorff A, Bulla S, Arndt S, Maier W, Laszig R, Beck R (2015). Radiologic results and hearing preservation with a straight narrow electrode via round window versus cochleostomy approach at initial activation. Otol Neurotol.

[15] Gazibegovic D, Bero EM (2017). Multicenter surgical experience evaluation on the Mid-Scala electrode and insertion tools. Eur Arch Otorhinolaryngol.

[16] Carlson ML, O'Connell BP, Lohse CM, Driscoll CL, Sweeney AD (2018). Survey of the American Neurotology Society on Cochlear Implantation: part 2, surgical and device-related practice patterns. Otol Neurotol.

[17] Connor SE, Holland NJ, Agger A, Leong AC, Varghese RA, Jiang D, Fitzgerald O'Connor A (2012). Round window electrode insertion potentiates retention in the scala tympani. Acta Otolaryngol.

[18] Tan H, Yao J, Li Y (2021). Radiological and audiological outcomes of the LISTENT LCI-20PI cochlear implant device. Otol Neurotol.

[19] Liu X, Xie L, Wang Y, Yang B (2019). Lower initial electrode impedances in minimally invasive cochlear implantation. Acta Otolaryngol.

[20] Hamerschmidt R, Schuch LH, Rezende RK, Wiemes GRM, Oliveira AKP de, Mocellin M (2012). A comparison between neural response telemetry via cochleostomy or the round window approach in cochlear implantation. Braz J Otorhinolaryngol.

[21] Cheng X, Wang B, Liu Y, Yuan Y, Shu Y, Chen B (2018). Comparable electrode impedance and speech perception at 12 months after cochlear implantation using round window versus cochleostomy: an analysis of 40 patients. ORL J Otorhinolaryngol Relat Spec.

[22] Briggs RJ, Tykocinski M, Xu J (2006). Comparison of round window and cochleostomy approaches with a prototype hearing preservation electrode. Audiol Neurootol.

[23] Zhou L, Friedmann DR, Treaba C, Peng R, Roland JT Jr (2015). Does cochleostomy location influence electrode trajectory and intracochlear trauma?. Laryngoscope.

[24] Schart-Morén N, Agrawal SK, Ladak HM, Li H, Rask-Andersen H (2019). Effects of various trajectories on tissue preservation in cochlear implant surgery: a micro-computed tomography and synchrotron radiation phase-contrast imaging study. Ear Hear.

[25] Driscoll CL, Carlson ML, Fama AF, Lane JI (2011). Evaluation of the hybrid-L24 electrode using microcomputed tomography. Laryngoscope.

[26] Sikka K, Kairo A, Singh CA (2017). An evaluation of the surgical trauma to intracochlear structures after insertion of cochlear implant electrode arrays: a comparison by round window and antero-inferior cochleostomy techniques. Indian J Otolaryngol Head Neck Surg.

[27] Kang BJ, Kim AH (2013). Comparison of cochlear implant performance after round window electrode insertion compared with traditional cochleostomy. Otolaryngol Head Neck Surg.

[28] Elafandi H, Khalifa MA, Elguindy AS (2020). Cochlear implantation outcomes with round window electrode insertion versus cochleostomy insertion. Int J Pediatr Otorhinolaryngol.

[29] Rajput M, Nilakantan A (2019). Functional outcomes in cochleostomy and round window insertion technique: difference or no difference?. Indian J Otolaryngol Head Neck Surg.

[30] Naderpour M, Aminzadeh Z, Jabbari Moghaddam Y, Pourshiri B, Ariafar A, Akhondi A (2020). Comparison of the pediatric cochlear implantation using round window and cochleostomy. Iran J Otorhinolaryngol.

[31] Korsager LE, Schmidt JH, Faber C, Wanscher JH (2018). Vestibular outcome after cochlear implantation is not related to surgical technique: a double blinded, randomized clinical trial of round window approach versus cochleostomy. Otol Neurotol.

[32] Cozma RS, Dima-Cozma LC, Rădulescu LM (2018). Vestibular sensory functional status of cochlear implanted ears versus non-implanted ears in bilateral profound deaf adults. Rom J Morphol Embryol.

[33] González-Navarro M, Manrique-Huarte R, Manrique-Rodríguez M, Huarte-Irujo A, Pérez-Fernández N (2015). Long-term follow-up of late onset vestibular complaints in patients with cochlear implant. Acta Otolaryngol.

[34] Esquia Medina GN, Borel S, Nguyen Y, Ambert-Dahan E, Ferrary E, Sterkers O, Grayeli AB (2013). Is electrode-modiolus distance a prognostic factor for hearing performances after cochlear implant surgery?. Audiol Neurootol.

[35] Davis TJ, Zhang D, Gifford RH, Dawant BM, Labadie RF, Noble JH (2016). Relationship between electrode-to-modiolus distance and current levels for adults with cochlear implants. Otol Neurotol.

[36] Martins Gde S, Brito Neto RV, Tsuji RK, Gebrim EM, Bento RF (2015). Evaluation of intracochlear trauma caused by insertion of cochlear implant electrode arrays through different quadrants of the round window. Biomed Res Int.

[37] Gstoettner W, Kiefer J, Baumgartner WD, Pok S, Peters S, Adunka O (2004). Hearing preservation in cochlear implantation for electric acoustic stimulation. Acta Otolaryngol.

[38] Briggs RJ, Tykocinski M, Stidham K, Roberson JB (2005). Cochleostomy site: implications for electrode placement and hearing preservation. Acta Otolaryngol.

[39] Todt I, Basta D, Ernst A (2008). Does the surgical approach in cochlear implantation influence the occurrence of postoperative vertigo?. Otolaryngol Head Neck Surg.

[40] Finley CC, Holden TA, Holden LK (2008). Role of electrode placement as a contributor to variability in cochlear implant outcomes. Otol Neurotol.

[41] Aschendorff A, Kromeier J, Klenzner T, Laszig R (2007). Quality control after insertion of the nucleus contour and contour advance electrode in adults. Ear Hear.

[42] Meshik X, Holden TA, Chole RA, Hullar TE (2010). Optimal cochlear implant insertion vectors. Otol Neurotol.

[43] Zelener F, Majdani O, Roemer A, Lexow GJ, Giesemann A, Lenarz T, Warnecke A (2020). Relations between scalar shift and insertion depth in human cochlear implantation. Otol Neurotol.

